# Editorial: Community series in novel biomarkers for predicting response to cancer immunotherapy: volume II

**DOI:** 10.3389/fimmu.2023.1328299

**Published:** 2023-12-11

**Authors:** Keyong Peng, Jinghua Pan

**Affiliations:** ^1^ Medical office of Panyu Compus, The First Affiliated Hospital of Jinan University, Guangzhou, China; ^2^ Department of General Surgery, the First Affiliated Hospital of Jinan University, Guangzhou, China

**Keywords:** cancer immunotherapy, biomarker, immune check inhibitor, response, prognosis

The field of cancer immunotherapy has revolutionized cancer treatment by harnessing the potential of the immune system to target and eliminate cancer cells. However, not all patients respond equally to immunotherapy or immunotherapy combination strategies, and predicting individual response remains a challenge. In recent years, there has been a growing interest in identifying biomarkers that can accurately predict a patient’s likelihood of responding to immunotherapy or immunotherapy combination strategies. This editorial summary aims to highlight the progress made in the research area of the *Community Series in Novel Biomarkers for Predicting Response to Cancer Immunotherapy: Volume II.*

One focus is set on the connection between pathological features and clinical outcomes of patients with cancer undergoing treatment with immune checkpoint inhibitors (ICB). Cheung et al. investigated whether the tissue protein expression of CD8, PD-L1, LAG-3, and STAT1 could predict responses to ICB in hepatocellular carcinoma (HCC). They found that immunohistochemical scoring of pre-treatment levels of LAG-3 and CD8 in the tumor microenvironment may help in predicting the benefits of ICB for patients with HCC, and immunohistochemistry-based techniques offer the advantage of being readily translatable in the clinical setting. For the clinical practice, Johansen et al. investigated the association between plasma YKL-40 levels and the clinical outcomes of patients with metastatic pancreatic cancer receiving ICIs and stereotactic body radiotherapy (SBRT). The results demonstrated that elevated baseline plasma YKL-40 was associated with lower overall survival. These findings suggested that YKL-40 could be a potential biomarker for predicting the response to ICIs and radiotherapy in patients with metastatic pancreatic cancer. Further research is required to validate these results. Moreover, Cui et al. were the first to compare the levels of endogenous circulating glucocorticoids in healthy people and patients with cancer. They found that an increase baseline endogenous circulating glucocorticoid has a comprehensive negative effect on immunosurveillance and the response to immunotherapy in patients with advancing cancer. In summary, these interesting findings about the relationship between clinical features and clinical markers in the context of tumor immunotherapy had been examined from different perspectives and demonstrate that easily to monitor immune and serum markers have valuable predictive value in cancer immunotherapies.

Moreover, some bioinformatics analyses for discovery of novel biomarkers of ICB in cancer based on big data had demonstrated promising findings. Li et al. collected HCC datasets from TCGA and GEO, including lysosome-related gene sets from AIMGO. They used machine learning to build risk models based on differentially expressed lysosome-associated genes in HCC and healthy tissues. The study concluded that lysosome-associated gene risk models can be used to predict prognosis in HCC and may provide new opportunities for chemotherapy and immunotherapy. RAMP3 may also be a potential target for HCC treatment. The exhausted CD8+ T (Tex) cells are a unique cell population of activated T cells that emerge in response to tumor antigens. Shi et al. demonstrated that Tex-related genes might provide accurate prediction for patients with HCC in clinical decision-making, prognostic assessment, and immunotherapy. Moreover, a study by Yang et al. identified diverse TPRGs (tumor-related prognostic genes) subtypes in lung adenocarcinoma (LUAD) and evaluated their prognostic value. The results evidenced that this novel stratification model based on TPRGs can accurately predict the prognosis of patients with LUAD and may serve as a predictive tool for treatment decisions. The F-box and WD repeat domain containing (FBXW) family of SCF E3 complexes has 10 members that are responsible for ubiquitination and degradation of substrate proteins involved in cell cycle regulation and tumorigenesis. Huang et al. reported that FBXW family genes were closely involved in immune components, such as immune score, immune subtypes, tumor-infiltrating lymphocytes, and immune checkpoints. Notably, FBXW1 as an oncogene and FBXW7 as a tumor suppressor gene also demonstrate opposite relationships on immune cells. Cui et al. also reported a novel signature based on nicotinamide metabolism-related genes that can be potentially used in clinical practice to evaluate prognosis and treatment efficacy in patients with breast cancer. Additionally, Brisebarre et al. found that the loss of the tumor suppressor FHIT in NSCLC leads to increased HER2 receptor activity in lung tumor cells, making them sensitive to anti-HER2 drugs. Subsequently, they conducted RNA sequencing analysis on tumor cells from NSCLC patients with diverse FHIT and pHER2 status to identify the transcriptomic signature associated with this phenotype. They found that ICI might not be a relevant option for patients with NSCLC and FHITlow/pHER2high tumors, and that anti-HER2 targeted therapy could be a good therapeutic alternative for this molecular subclass with poorer prognosis. These studies demonstrate several emerging biomarkers and gene models for predicting response to ICB. Further study of the clinical application of these biomarkers is required.

Importantly, there were some publications on this research field that include important clinical results based on ICB. According to Chen et al. immunotherapy with single-agent chemotherapy as a second- or later-line treatment is safe, effective, and tolerable for metastatic NSCLC. Extracellular vesicle (EV) markers can be used as predictive markers of efficacy in patients with metastatic NSCLC treated with immunotherapy and chemotherapy to help monitor treatment efficacy and guide treatment decisions. A meta-analysis by Luo et al. reported that PD-1/PD-L1 inhibitors in combination with chemotherapy significantly improves PFS and OS in patients with extensive-stage small-cell lung cancer (ES-SCLC) without increasing the overall incidence of treatment-related adverse events (TRAEs). These studies highlighted that combined immunotherapy is a trend for clinical application in the future, which will further improve the clinical effect of ICB in patients with cancer.

In conclusion, the research and application of novel biomarkers for predicting responses to cancer immunotherapy have significantly advanced the field. Immune-related biomarkers, immune cell profiling, and molecular biomarkers have all shown promise in accurately predicting individual response to immunotherapy. Incorporating these biomarkers into clinical practice has the potential to guide treatment decisions, enhance patient outcomes, and ultimately improve the effectiveness of cancer immunotherapy.

## Author contributions

KP: Writing – original draft. JP: Writing – review & editing.

